# Measurement of Variceal Pressure with a Computerized Endoscopic Manometry: Validation and Effect of Propranolol Therapy in Cirrhotic Patients

**DOI:** 10.1371/journal.pone.0056332

**Published:** 2013-02-19

**Authors:** De-Run Kong, Chao Zhang, Lei Zhang, Jing-Guang Wang, Zhuang Xiong, Pan Li, Jian-Ming Xu

**Affiliations:** 1 Department of Gastroenterology, First Affiliated Hospital of Anhui Medical University, Hefei, Anhui, People’s Republic of China; 2 Department of Surgery, First Affiliated Hospital of Anhui Medical University, Hefei, Anhui, People’s Republic of China; 3 Department of Radiology, First Affiliated Hospital of Anhui Medical University, Hefei, Anhui, People’s Republic of China; Cardiff University, United Kingdom

## Abstract

**Background and Purpose:**

Recently, we invented a computerized endoscopic balloon manometry (CEBM) to measure variceal pressure (VP) in cirrhotic patient. The purpose of this study was to evaluate the reliability and feasibility of this method, and whether this technique provided further information to pharmacological therapy.

**Patients and Methods:**

VP measurements were performed in 83 cirrhotic patients and compared with HVPG as well as endoscopic bleeding risk parameters. Furthermore, VP was assessed before and during propranolol therapy in 30 patients without previous bleeding.

**Results:**

VP measurements were successful in 96% (83/86) of all patients. Of the 83 patients, the VP correlated closely with the HVPG (*P*<0.001). The presence of red colour signs and the size of varices were strongly associated with VP. Patients with previous bleeding had higher VP than those who had not yet experienced bleeding. In univariate analysis, the level of VP, the size of varices, and red color signs predicted a higher risk of bleeding. The multiple logistic regression model revealed that VP was the major risk factor for bleeding. In 30 patients receiving propranolol, VP significantly decreased from 21.1±3.5 mmHg before therapy to 18.1±3.3 mmHg after 3 months and to 16.3±4.0 mmHg after 6 months. Comparing the mean decrease in VP with that in hepatic venous pressure gradient (HVPG), the decrease in VP was more obvious than HVPG response to propranolol.

**Conclusions:**

This study showed that CEBM is safe and practical to assess VP in cirrhotic patient. It has the potential to be used as a clinical method to assess the risk of variceal bleeding and the effects of pharmacological therapy.

**Trial registration:**

Effect of vasoactive drugs on esophageal variceal hemodynamics in patients with portal hypertension. Chinese Clinical Trial Registry –TRC-08000252.

## Introduction

Previous studies have revealed that variceal pressure VP is a major predictor of variceal bleeding risk and of the response to pharmacological therapy in patients with portal hypertension [1]–[4]. VP can be measured noninvasively at endoscope by both pressure gauge and transparent balloon in earlier study [5]–[6]. Noninvasive techniques assume that varices behave as an elastic structure because of their thin walls and lack of external tissue support; thus, the pressure needed to compress a varix equals the pressure inside the varix [7]. However, it was often difficult to determine the precise moment when the variceal wall started to collapse (which can be sensed by pressure gauges or under direct vision using transparent balloons), which left these method significantly observer-dependent and could affect judgment of the VP values. Few years later, several new devices combining endosonography and manometry has been designed by power Doppler for monitoring the blood flow in esophageal varices, instead of the direct vision of variceal compression during balloon manometry [8]–[9]. VP is considered as the pressure needed by the balloon to cause the disappearance of flow inside the varix during its compression. This modified method has suggested its reliability and accuracy in vitro studies, but clinical studies are still lacked. Recently, we invented a computerized endoscopic balloon manometry (CEBM), with computer vision to determine the moment of variceal wall collapse instead of direct vision using in the traditional method [10]. However, that study only reported a univariate association because the sample size and event numbers were small, the clinical reliability and feasibility of this method in cirrhotic patient and esophageal varix needed further validate. We then prospectively accumulated sufficient cases to verify the previous results, by conducting a multivariate analysis. Therefore, in the study, we aimed to determine whether the CEBM was a safe and reliable method to evaluate VP, an important parameter related to the risk of variceal bleeding, and to evaluate the effectiveness of pharmaceutical therapy in patients with portal hypertension.

## Patients and Methods

### Ethics Statement

Clinical and laboratory examinations were performed after obtaining informed written consent from all patients and approval from the Ethics Committee in Anhui Medical University.

### Patients

Between January 2006 and June 2010, 83 cirrhotic patients were enrolled to the group and agreed to participate in the study (Checklist S1). Liver cirrhosis had been diagnosed in the patients according to clinical, biochemical, endoscopic, histological, or ultrasonographic criteria. Hepatic hemodynamic and endoscopic measurements were considered as a standard diagnostic work-up in the patients, included an upper gastrointestinal endoscopy, as well as a transjugular liver biopsy with measurement of the hepatic venous pressure gradient (HVPG). VP measurement was performed at the time of upper gastrointestinal endoscopy. Patients with portal vein thrombosis, treatment with beta-blockers, previous endoscopic treatment of varices (sclerotherapy or endoscopic band ligation), multifocal hepatocellular carcinoma, severe clotting defects, hepatic encephalopathy grade III and IV, previous surgical portosystemic shunts or intrahepatic portosystemic stent-shunt were excluded from the study.

**Figure 1:**
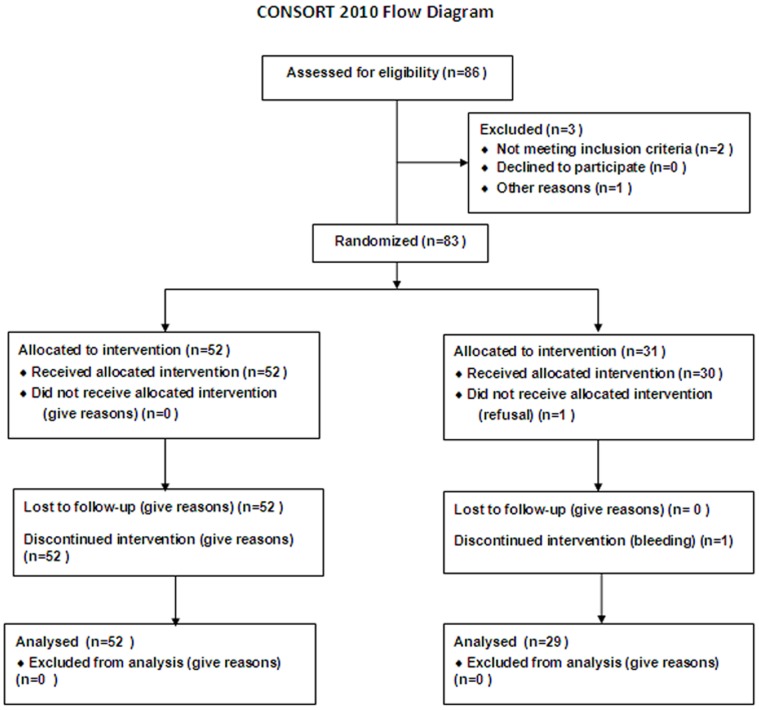
Enrollment of patients with cirrhosis and portal hypertension.

Fifty-two (63%) patients had already experienced a variceal hemorrhage. The majority of 52 patients who experienced bleeding visited our hospital within 1 week of bleeding symptoms. Before VP measurement vasoactive therapeutic agents were discontinued at least 5 days. Of these 31 patients (37%) had never experienced variceal hemorrhage, 30/83 patients were treated prophylactically with propranolol. VP was measured before, and 3 months after and 6 months after starting the treatment in the patients (Fig. 1). A 25% reduction of the heart rate or maximal tolerated doses of propranolol was the standard goal to monitor the effectiveness of the propranolol therapy. At the same time, VP and HVPG were assessed before and after treatment. A decrease in HVPG <12 mmHg or >20% from baseline was considered as criteria of clinical response [11].

### Measurement of Variceal Pressure

**Figure 2:**
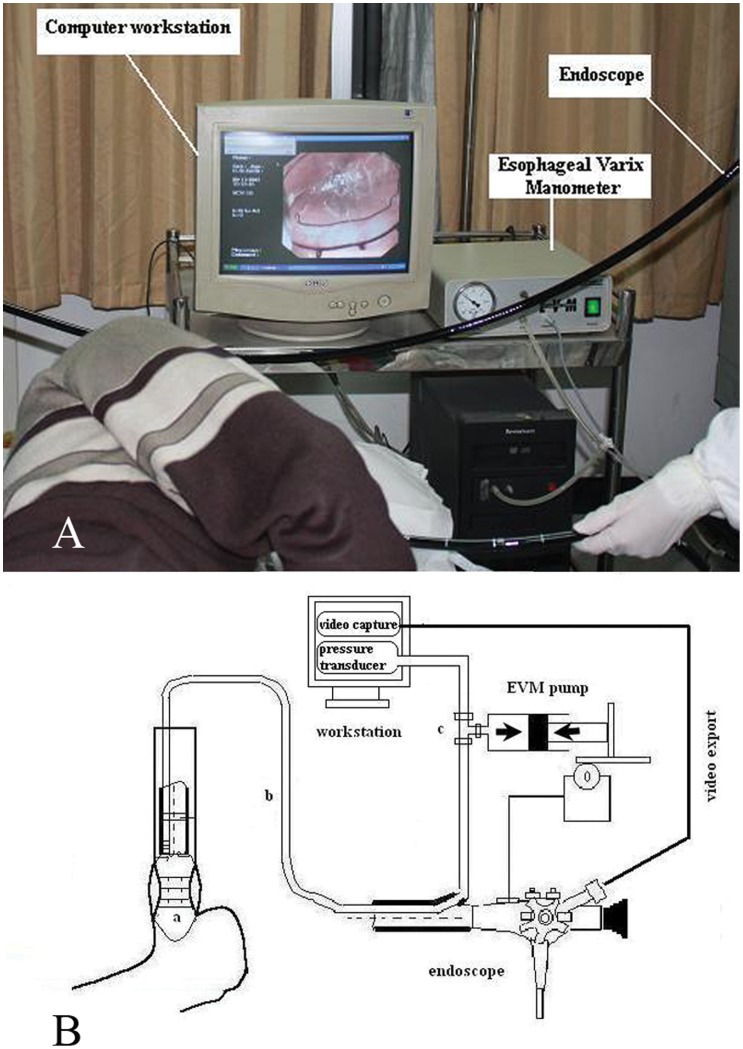
Schematic representation of computerized endoscopic balloon manometry (CEBM). The endoscopic balloon (a), introduced into the distal esophagus, is connected via a catheter (b) to integral air pump for esophageal variceal manometry connected to the CEBM workstation by the triple stopcock. The CEBM simultaneously records VP and endoscopic image.

Measurements of VP were performed at endoscopy using a previously described CEBM technique which was developed by our group [10]. The CEBM was composed of an esophageal variceal manometer (EVM; Esophageal Varix Manometer; Treier Endoscopie AG, Beromünster, Switzerland) and a PC workstation, which recorded the pressure signal from the manometry and digital video signal from the endoscope simultaneously (Fig. 2).

**Figure 3:**
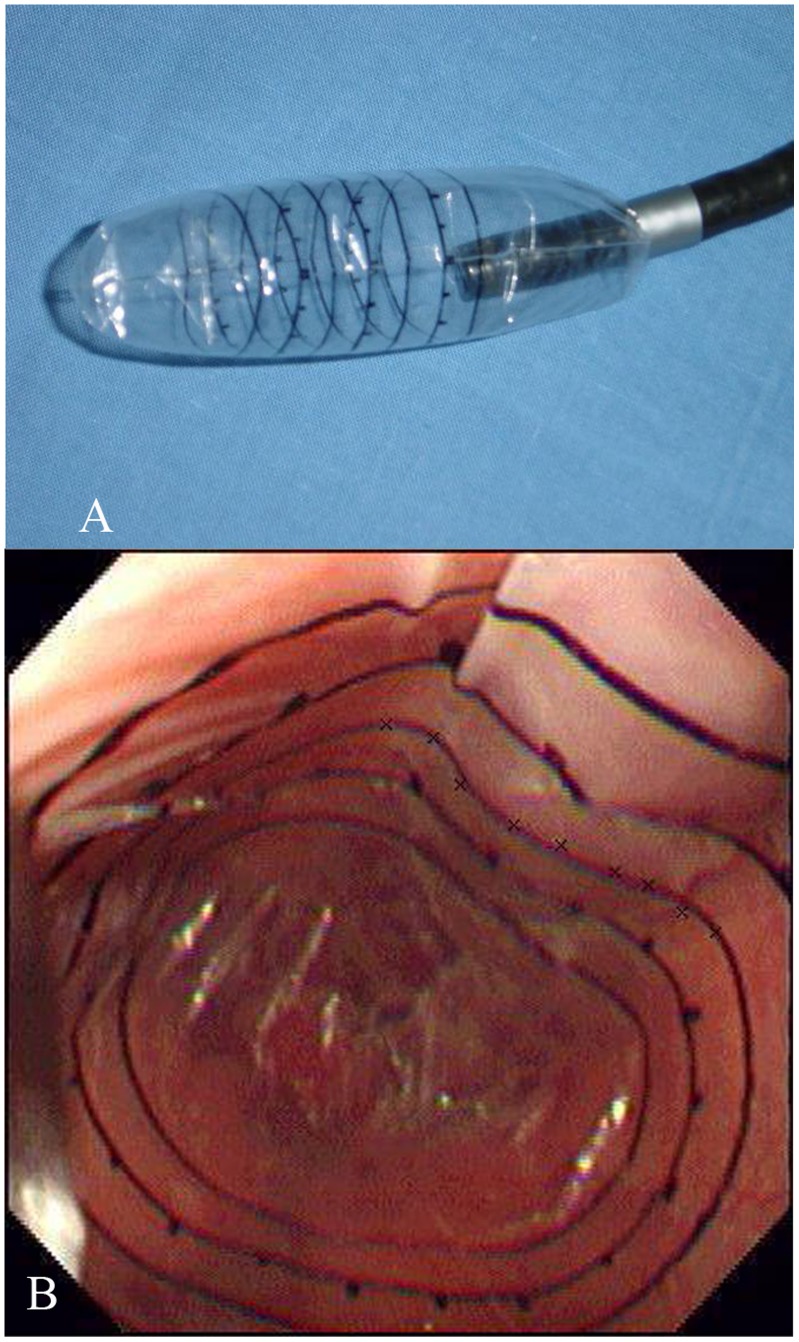
Transparent balloon fixed at the tip of endoscope. **a** The circular lines on the balloon were used as the variceal markers to monitor varix flatting. The scales in the lines (5-mm intervals) were used to estimate the variceal size. **b** Variceal markers highlighted the regular curve of the underlying varices.

This CEBM used a transparent balloon fixed to the distal end of endoscope (Fig. 3). To minimize esophageal tonus and peristalsis, all patients received premedication with 5 mg diazepam and 20 mg *n*-butylscopolamine intravenously. When the endoscope was inserted into the stomach, the balloon was partially inflated in order to unfold it. After this was done satisfactorily, the balloon was then withdrawn into the distal esophagus. The largest varix situated above the cardia was chosen for VP measurement. Once the varices were clearly visible, the balloon was slowly inflated until the wall of the varix flattening completely, which appeared as decrease in height of the varix. Because both the balloon pressure from the manometry and the endoscopic video were simultaneously recorded by CEBM, when the varices began to flatten, the balloon pressure was equal to the VP which was recorded by the workstation. The method that the investigators determined the VP was to identify the decrease in height of the varix, which was read from the endoscopic videotape. To eliminate observer bias, determination of the timing of balloon flattening was conducted by two independent investigators for each measurement blinded to the pressure. The mean of five satisfactory measurements was used to calculate VP and intra- as well as inter-observer agreement.

After VP measurement, the size of the varix was estimated in the absence of peristaltic waves, by comparing the varix with the scales in the balloon variceal markers (5-mm intervals). The maximal size of the varices and the red colour signs were recorded as proposed by the Japanese Research Society for portal hypertension [12].

### Measurement of HVPG

Hemodynamic studies were performed in all patients after endoscopic procedures, and within two days from endoscopic investigations. After an overnight fasting period, a 5F Cobra catheter was introduced into the main right hepatic vein under fluoroscopic guidance for measurements of wedged (occluded) and free hepatic venous pressures under local anesthesia. HVPG was determined by the difference between the wedged hepatic venous pressure and the free hepatic venous pressure. Pressure tracings were maintained stable at least 30 seconds to be considered satisfactory. A mean of three readings was taken in each patient. If there was a difference of more than 1 mm Hg between the readings, all the recordings were discarded and fresh readings were taken. Transjugular liver biopsy was performed in each patient at the end of the hemodynamic procedures.

### Statistical Analyses

Quantitative data were expressed as mean ± standard deviation (SD). Each continuous parameter was analyzed with the two-sample Student *t*-test. Paired t-tests were used to examine change from baseline to follow-up. Categorical data were examined using McNemar’s test. The correlation between variables was analyzed using the Spearman correlation test. The relationship of the difference between VP and Child-Pugh score was checked by ANOVA, with a least-significant test *post hoc* adjustment. Univariate analysis and forward stepwise multivariate logistic regression were used to analysis the risk factors for initial variceal bleeding. Statistical analysis was done using the SPSS 12.0 software package. Statistical significance was defined as *P*≤0.05.

## Results

### Clinical Characteristics of Patients

Of the 83 patients included, there were 62 males and 21 females, with a mean age of 47.2±13.1 yr. The classification of the patients according to etiology was hepatitis B virus (HBV) infection in 67 (81%), alcohol taking in 5(6%), primary biliary cirrhosis in 11 (13%). Twenty-seven (33%) patients belonged to Child-Pugh class A, 51 (61%) to class B, and 5 (6%) to class C. The patients had *F*_1_(n = 3), *F*_2_ (n = 15) and *F*_3_ (n = 65) esophageal varices according to the Japanese Research Society for Portal Hypertension. The mean VP value was 24.6±4.2 mmHg. More details on the patients were presented in Table 1.

**Table 1:** Demographic and clinic profile of the study population.

Total number of patients	83
Age (yr)	47.2±13.1
Sex	
Male	62 (74.7%)
Female	21 (25.3%)
Etiology	
Viral	67 (80.7%)
Alcohol	5 (6.0%)
primary biliary cirrhosis	11 (13.3%)
Child-Pugh score	
A	27 (32.5%)
B	51 (61.5%)
C	5 (6.0%)
Ascites	
Yes	50 (60.2%)
No	33 (39.8%)
Bleeding	
Yes	52 (62.7%)
No	31 (37.3%)
Size of varices, mm	8.27±2.33
Red color sign present	
Yes	44 (53%)
No	39 (47%)
Varix grade	
* F_1_*	3 (3.6%)
* F_2_*	15 (18.1%)
* F_3_*	65 (78.3%)
Variceal pressure, mm Hg	24.6±4.2
HVPG (mm Hg)	18.3±3.7

### Evaluation of VP Measured by CEBM

The study was performed in 86 patients initially. One patient was excluded because of hard retching and uncontrolled coughing during intubation. Of the remaining 85 patients, adequate VP measuring could not be obtained from two patients because of too small varices. Therefore, 83 satisfactory VP were obtained in 415 measurements. Intra- and inter-observer agreement was determined by calculation of correlation coefficient. The correlation coefficient for intra-observer reliability of VP measurement was 0.95. The correlation coefficient for inter-observer reliability of VP measurement was 0.97. No patients experienced variceal bleeding in the VP measuring session.

### Comparison of VP and HVPG

**Figure 4:**
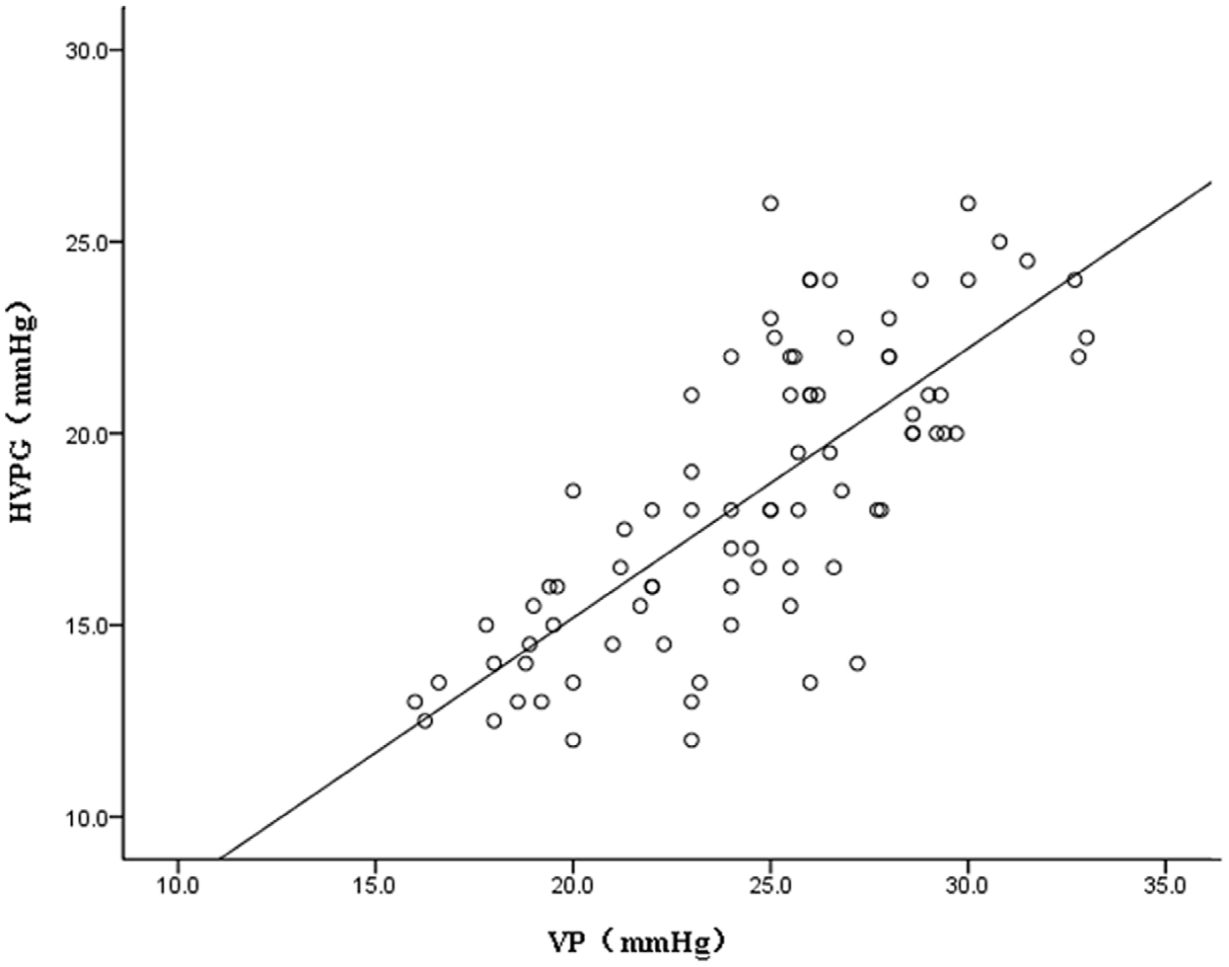
Correlation between the HVPG and the VP measured by CEBM in 83 patients with cirrhosis and esophageal varices.

Both VP and HVPG measurements were obtained in 83 patients. VP measured by CEBM was 24.6±4.2 mmHg, and HVPG was 18.3±3.7 mmHg. There was a significant correlation between VP and HVPG (*r* = 0.75, *P*<0.001, Fig. 4).

### VP in Relation to Hemorrhage

**Figure 5:**
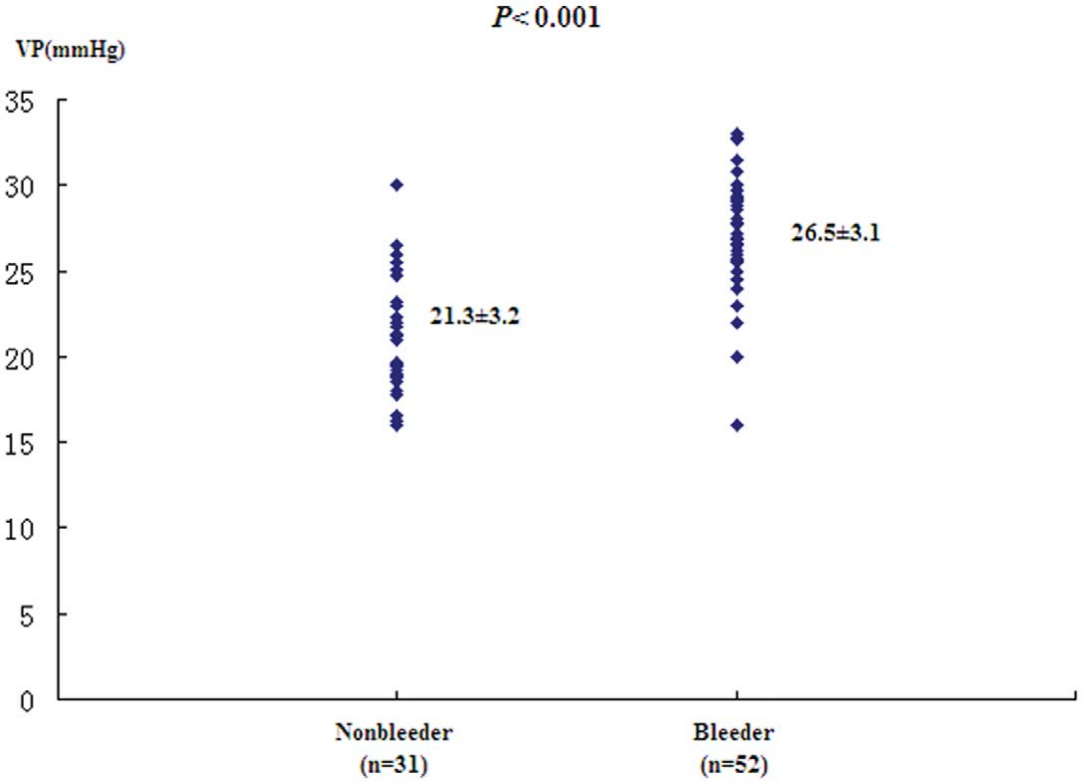
VP levels (mm Hg) measured by CEBM in bleeder and nonbleeder patients with cirrhosis and esophageal varices.

VP obtained with CEBM in 52 patients, who had experienced variceal bleeding before the measurement was 26.5±3.1 mmHg, as compared with 21.3±3.2 mmHg in 31 patients who had never bled (*P*<0.001, Fig. 5). In univariate analysis, the level of VP (*P* = 0.001), the size of varices (*P* = 0.006), and the endoscopic red color sign on the variceal wall (*P* = 0.012) predicted a higher risk of variceal hemorrhage. With all variables in the univariate analysis, a multiple logistic regression model revealed that VP was the major risk factor for initial variceal bleeding (*OR* = 1.444, 95%CI: 1.223∼1.704, *P*<0.001).

### Comparison of VP with Size of Varices, Presence of Red Colour Signs, Child-Pugh Score

**Figure 6:**
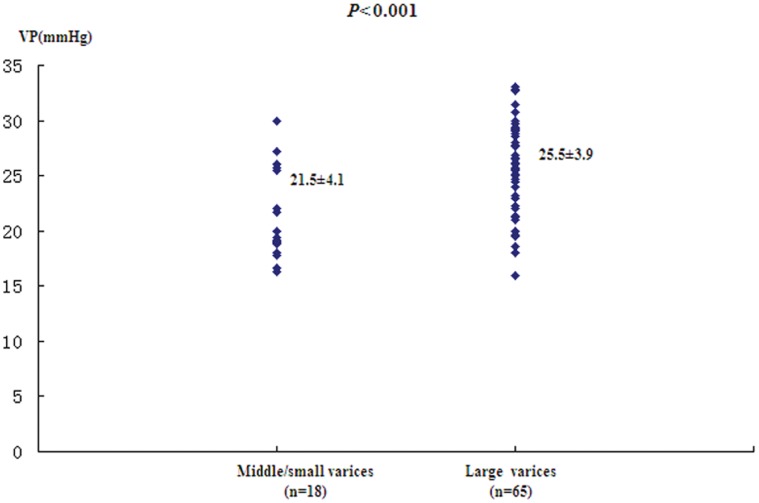
VP levels (mm Hg) measured by CEBM in patients with middle/small and large esophageal varices.

**Figure 7:**
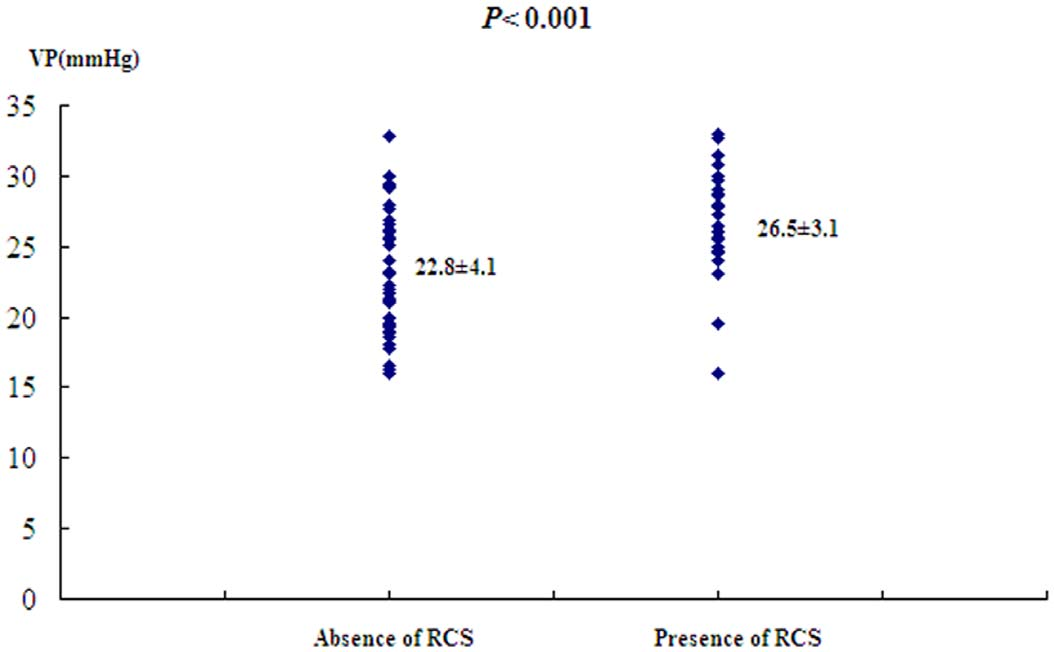
VP levels (mm Hg) measured by CEBM in correlation with the presences absence of red colour signs.

Of the 83 patients, VP was higher in large varices (*n = *65) than in middle/small ones (*n = *18). The differences were highly significant (25.5±3.9 *vs*. 21.5±4.1 mmHg, *P*<0.001, Fig. 6). VP were also significantly higher in patients with red colour signs: 26.5±3.1 mmHg (*n* = 44) than in those without red colour signs: 22.8±4.1 mmHg (*n* = 39, *P*<0.001, Fig. 7). The Child-Pugh score did not correlate with VP (*F* = 1.19, *P* = 3.15). The mean VP in patients in Child-Pugh A cirrhosis (*n* = 27) was 24.6±3.4 mmHg, whereas in Child-Pugh B (*n* = 51) and C (*n* = 5), it was 24.2±4.3 and 27.2±5.0 mmHg, respectively.

### Evaluation of the CEBM in Patients Receiving Propranolol

Thirty patients in the study group without any previous variceal bleeding received prophylactic treatment with propranolol (30 to 80 mg/day, median: 60 mg). As shown in table 2, propranolol administration caused a significant reduction in VP (from 21.1±3.5 mmHg to 18.1±3.3 mmHg after 3 months; *P*<0.0001 and to 16.3±4.0 mmHg after 6 months; *P*<0.0001). The mean decrease was 14.0±7.2% after 3 months and 23.0±13.9% after 6 months. At the same time, the propranolol administration also caused a significant reduction in HVPG from 15.7±2.9 mmHg to 13.7±2.4 mmHg after 3 months and to 12.7±2.4 after 6 months in patients responding to therapy (*P*<0.0001). The mean HVPG decrease was 11.8±6.0% after 3 months and 19.1±9.9% after 6 months. However, comparing the mean decrease in VP with that in HVPG after 3 months (*t* = 2.820, *P* = 0.007) and 6 months (*t* = 2.571, *P* = 0.013), the mean decrease in VP was more obvious than HVPG response to propranolol. According to maximal tolerated dosage or decrease of the heart rate 25% from baseline, 21 out of 30 patients responded to propranolol therapy as assessed by the decrease in the HVPG to 12 mm Hg or less or a decrease in the initial HVPG of more than 20%. Variceal size decreased during prophylactic treatment with propranolol, from 7.24±2.44 mm at initiation to 6.83±1.98 mm after 6 months. Statistical significance of the decrease in variceal size had no difference (*P*>0.05). The disappearance of red colour signs was observed in two patients received propranolol therapy after 6 months, and statistical significance had no difference (*P* = 0.50). However, despite continued intake propranolol of a dosage of 80 mg/day until heart rate was less than 55 beats per minute, two out of thirty patients did not respond to the therapy as evaluated by an increasing VP. One of them experienced a variceal hemorrhage and received endoscopic variceal sclerotherapy immediately.

**Table 2:** Changes in variceal pressure, hemodynamics, endoscopic findings under propranolol administration.

variable	Baseline		After 3 months			After 6 months		
	mean±SD	95% CI	mean±SD	95% CI	*P*_1_ value	mean±SD	95% CI	*P*_2_ value
VP, mm Hg	21.1±3.5	20.0–22.5	18.1±3.3	16.8–19.3	0.000*	16.3±4.0	14.8–17.8	0.000*
HVPG, mm Hg	15.7±2.9	14.6–16.9	13.7±2.4	12.8–14.6	0.000*	12.7±2.4	11.7–13.6	0.000*
HR, beats/min	84±10	80.4–87.6	65±9	61.8–68.2	0.000*	63±8	60.1–65.9	0.000*
Size of varices, mm	7.24±2.44	6.31–8.17	7.03±1.94	6.30–7.77	0.297	6.83±1.98	6.07–7.58	0.222

NOTE: HR = heart rate. *P*_1_ Baseline/after 3 months comparisons, *P*_2_ Baseline/after 6 months comparisons.

* *P* values for trends.

## Discussion

Previous studies have identified VP as a prognostic indicator of the bleeding risk and of the response to pharmacological therapy in patients with both cirrhosis and noncirrhotic portal hypertension [2]–[3]. The “gold standard” is the measurement of intravariceal pressure by direct puncture of the varix with a thin needle. In this day and age of band ligation, it is no longer considered acceptable or ethical to perform variceal needle puncture to measure intravariceal pressure. For this reason, at least four noninvasive methods have been developed (mainly endoscopic pressure sensitive gauges and balloon manometry) have been developed to measure intravariceal pressure [5]–[6], [8]–[9]. Noninvasive endoscopic techniques for assessing VP based on force balance laws that at the initiation of variceal flattening, the pressure required to collapse varix is equal to the intravariceal pressure [7]. All these attempts at measuring intravariceal pressures noninvasively were found to be technically successful in vitro, but have not been disseminated widely to clinicians and remain mostly a research tool. Because, in practice it is often quite difficult to decide the point of variceal flattening depends on the visual interpretation of the degree of variceal compression by the balloon, which would overestimate VP value by the observer [8].

In our recent study, we invented a CEBM technique in which computer vision was used to determine the point of variceal flattening during inflation and deflation of the balloon, even with a small varix where there was only a slight change in variceal wall. With a small number of cirrhotic patients, the preliminary results had indicated that CEBM might contribute measurement of VP [10].

Consistent with our previous studies, we found that there was a good correlation between VP and HVPG in the large population with portal hypertension and varices. The correlation coefficients for intra-observer and inter-observer showed that the results of CEBM were also reliable in VP measurement. Furthermore, in contrast to traditional VP measuring techniques, CEBM may be able to measure pressure in a small varix as accurately as in a large varix ideally. Therefore, the present study provides further evidence validating the use of CEBM to measure VP in cirrhotic patients. In addition, an excellent correlation between VP measured by CEBM and endoscopic features of variceal bleeding risk (variceal size and presence of red color signs) was found, and VP was higher in patients with a history of variceal bleeding as compared to those who did not experience a bleeding. Moreover, with all variables in the univariate analysis, a multiple logistic regression model revealed that VP was the major risk factor for initial variceal bleeding in our group of patients. These results strongly suggest that CEBM may be useful to identify patients at a high risk for bleeding from the varices, and may be alternative to HVPG measurements. These patients with the high-risk factors may need longer-lasting therapeutic options than the initial treatment. These options may include the endoscopic variceal ligation, endoscopic sclerotherapy to eradicate any varices. In all patients, CEBM can be safely used without serve adverse effects of variceal bleeding during routine endoscopic evaluation, and might become a valuable research tool.

Our study also evaluated whether CEBM technique allowed assessing the effects of propranolol therapy in VP. The present evaluation showed that propranolol caused a significant decrease in VP, associated with a reduction in HVPG and endoscopic features of variceal bleeding risk (variceal size and presence of red color signs). Moreover, the mean decrease in VP of 14.0±7.2% after 3 months and 23.0±13.9% after 6 months of propranolol treatment was more obvious than that in HVPG (11.8±6.0% after 3 months, *t* = 2.820, *P* = 0.007 and 19.1±9.9% after 6 months, *t* = 2.571, *P* = 0.013; respectively). The results show that the prognostic value of the VP in patients receiving pharmacological therapy with propranolol is as powerful as that of the HVPG response. In our study, the mean dosage of propranolol given was lower than most studies. However, the low dosage of propranolol in current study is expected because it is well know that the metabolism of this drug is different between Asian and European patients. In previous study, Lay et al. found that the mean daily dosage of the propranolol was 68.2±32.8 mg, which was sufficient to reduce the heart rate by 25%. It is therefore possible that a lower dosage of propranolol to reach a target heart-rate reduction of 25% would have enough power to result in a lower bleeding rate in a Chinese population [13].

However, the CEBM technique has several limitations. First, it is imperative that the patients could be able to cooperate. Theoretically, the presence of artifacts caused by esophageal peristalsis, coughing and retching probably affect the accuracy of VP measurement. This difficulty have been overcome in the study, in which the VP measured by CEBM was achieved in 97% (83/86) of patients studied because the course was performed under sedation and VP was correctly measured between peristaltic. We also notice that 2% (2/86) of VP measurements was technically difficult and time consuming in patients with some very small varices, because of difficulties achieving satisfactory tracings. Though this method was applicable for the patients with variceal size ranging from *F*_1_ to *F*_3_, the minimum size of the varix for the application of this methodology is great than or equal to 3 mm in our group of patients. However, in patients with very small varices, VP measurement is probably not very important as bleeding is rare.

In conclusion, the present study further confirms that the CEBM technique allowed an accurate estimation of VP in patients with portal hypertension, which showed a close relation with parameters that were associated with an increased risk of variceal hemorrhage. CEBM technique has the potential to be used as a clinical method to predict the risk of initial variceal bleeding and to monitor pharmacologic therapy.

## Supporting Information

Checklist S1 CONSORT 2010 checklist of information to include when reporting a randomised trial.
(DOC)
